# Solvent Regulation in Layered Zn-MOFs for C_2_H_2_/CO_2_ and CO_2_/CH_4_ Separation

**DOI:** 10.3390/molecules30051171

**Published:** 2025-03-05

**Authors:** Xingyao Zhao, Xiaotong Chang, Caixian Qin, Xiaokang Wang, Mingming Xu, Weidong Fan, Qingguo Meng, Daofeng Sun

**Affiliations:** 1 School of Materials Science and Engineering, China University of Petroleum (East China), Qingdao 266580, China; 2214011219@s.upc.edu.cn (X.Z.); 2214010616@s.upc.edu.cn (X.C.); 2214011205@s.upc.edu.cn (C.Q.); xumingming.slyt@sinopec.com (M.X.); dfsun@upc.edu.cn (D.S.); 2 College of Chemical Engineering and Environmental Chemistry, Weifang University, Weifang 261061, China; mengqg@wfu.edu.cn

**Keywords:** metal–organic framework, C_2_H_2_/CO_2_ separation, CO_2_/CH_4_ separation

## Abstract

The development of alternative adsorptive separation technologies is extremely significant for the separation of C_2_H_2_/CO_2_ and CO_2_/CH_4_ in the chemical industry. Emerging metal–organic frameworks (MOFs) have shown great potential as adsorbents for gas adsorption and separation. Herein, we synthesized two layered Zn-MOFs, UPC-96 and UPC-97, with 1,2,4,5-tetrakis(4-carboxyphenyl)-3,6-dimethylbenzene (TCPB-Me) as a ligand via the solvent regulation of the pH values. UPC-96 with a completely deprotonated ligand was obtained without the addition of acid, exhibiting two different channels with cross-sectional sizes of 11.6 × 7.1 and 8.3 × 5.2 Å^2^. In contrast, the addition of acid led to the partial deprotonation of the ligand and afforded UPC-97 two types of channels with cross-sectional sizes of 11.5 × 5.7 and 7.4 × 3.9 Å^2^. Reversible N_2_ adsorption isotherms at 77 K confirmed their permanent porosity, and the differentiated single-component C_2_H_2_, CO_2_, and CH_4_ adsorption isotherms indicated their potential in C_2_H_2_/CO_2_ and CO_2_/CH_4_ separation.

## 1. Introduction

In the face of energy shortages and environmental crises, increasing attention has been paid to cleaner and more economical energy resources such as natural gas, biogas, shale gas, and coalbed methane [1,2,3]. The main component of these gas resources is methane (CH_4_), and they usually contain impurity gas, such as carbon dioxide (CO_2_), which may lead to the corrosion of pipelines during conveyance [4,5,6]. Therefore, it is important to selectively capture CO_2_ and realize the efficient purification of CH_4_. Acetylene (C_2_H_2_) is a significant raw material in the petrochemical industry, which has been widely used as fuel in welding and to produce fundamental chemicals like polyvinyl chloride, butadiene, and acrylic acid derivatives, as well as synthetic materials like rubber, fiber, and plastic [7,8,9]. Nowadays, the main source of C_2_H_2_ is the partial combustion of CH_4_ and cracking of hydrocarbons, which inevitably generate CO_2_ as a by-product [10,11,12]. Due to having similar boiling points (189.3 K for C_2_H_2_ and 194.7 K for CO_2_) and the same kinetic diameter (3.3 Å), it is a big challenge to remove CO_2_ and acquire high-purity C_2_H_2_ that meets the needs of the chemical industry. Therefore, it is necessary to capture and remove CO_2_ for energy gas storage and industrial raw gas purification. Carbon capture and storage (CCS) covers a range of technologies that will be crucial in supporting these separations. CCS technologies have matured significantly, with over 30 commercial facilities operating globally as of 2023, and the total CO_2_ capture capacity of operational projects has exceeded 40 million tons per annum (Mtpa), with many more projects under development [13].

Traditional separation processes mainly include cryogenic distillation and solvent absorption. Cryogenic distillation is energy-intensive due to the huge amount of energy required to realize phase changes, and solvent absorption is environmentally unfriendly due to the use of massive amounts of toxic solvents [14]. In comparison, adsorbent-based gas separation represents an energy-efficient and environmentally friendly separation technology [15,16,17,18,19,20,21,22]. So far, various adsorbents have been researched and developed, among which metal–organic frameworks (MOFs) are outstanding due to their high specific surface area, tunable pore size, and facile modification with functional groups in comparison with traditional adsorbents like zeolite, molecular sieve, and activated carbon [23,24,25,26,27,28,29]. Hence, MOFs are regarded as efficient and adaptable adsorbents with low energy consumption, and great progress has been made in the application of gas separation [30,31,32,33,34,35,36]. As structure determines performance, the regulation of an MOF’s structure can effectively optimize the gas adsorption and separation performance. For example, Zhou et al. investigated the effect of solvent proton-supplying/accepting capacity on the structure and CO_2_ adsorption performance of Mg-MOF-74 through the regulation of the polarity of the reaction solvent [37]. The highest performing sample was synthesized in an N,N’-dimethylformamide and methanol environment with a high CO_2_ adsorption capacity of 7.38 mmol/g. Lewiński et al. reported the controlled construction of two-dimensional (2D) MOFs through solvent-templated growth and obtained a series of 2D Cu(II)–carboxylate MOFs with various stacking modes and distances with a diffusion-controlled MOF deposition approach in different solvents [38]. Further research on the gas adsorption properties demonstrated the crystal size had a remarkable effect on the nitrogen (N_2_) adsorption capacity, which may be related to the limited number of pore entrances in the bigger flake-shaped crystals. Jeong et al. developed a solvent-assisted reversible strategy to modulate the interpenetration form, in which interpenetration was accomplished with protic solvents of small molecular sizes, such as water, methanol, and ethanol, and non-interpenetration was achieved using pyridine with Lewis basicity [39]. Compared to the non-interpenetrated MOF-143(NI) with a large pore size, the interpenetrated MOF-14(I) with narrow pores exhibited a higher ethane and ethylene uptake capability, as well as higher levels of ethane selectivity. Therefore, studies on solvent regulation in MOFs to achieve the effective optimization of specific gas adsorption and separation performance are attractive, and there is still much room for exploration.

Herein, we synthesized two kinds of 2D layered Zn-MOFs, UPC-96 and UPC-97, with methyl modified tetracarboxylic 1,2,4,5-tetrakis(4-carboxyphenyl)-3,6-dimethylbenzene (TCPB-Me) as a ligand. The solvent regulation of the pH value led to different coordination modes of the ligand and thus afforded two distinct frameworks. The difference in the porosity of UPC-96 and UPC-97 resulted in obviously differential levels of adsorption and separation performance. UPC-96 performed better than UPC-97 in terms of Brunauer–Emmett–Teller (BET) surface area, pore volume, adsorption capacity, enthalpy, and selectivity.

## 2. Results and Discussion

### 2.1. Crystal Structure

**Figure 1:**
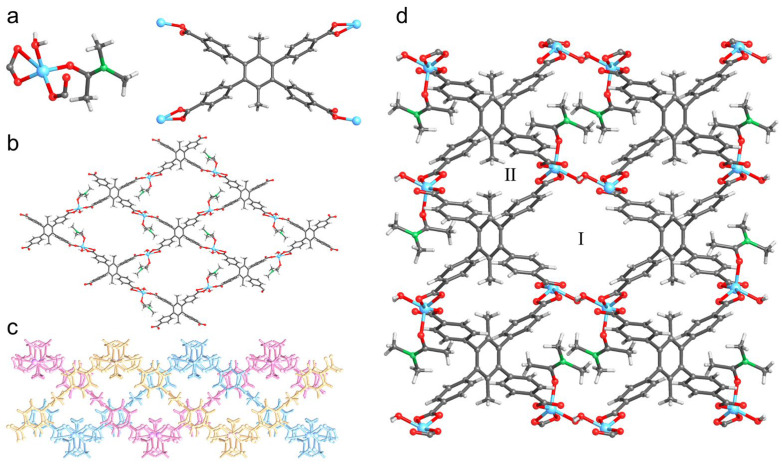
Crystal structure of UPC-96: (**a**) Coordination mode of Zn^2+^ and ligand; (**b**) Single layered framework with *sql* topology; (**c**) 2D layered unit with three layers; (**d**) 3D framework with two channels. Zn, sky-blue; C, gray; N, green; O, red; and H, pale.

Colorless rod-shaped crystals of UPC-96 were obtained by a solvothermal reaction following the method in Section 3.3. Single-crystal X-ray diffraction (SCXRD) measurements indicate that UPC-96 crystallizes in the monoclinic *P*2_1_/c space group. The asymmetric unit contains one Zn^2+^ ion, one half of a completely deprotonated ligand, one water molecule, and one N,N’-dimethylacetamide (DMA) molecule. Each Zn^2+^ ion is coordinated with three oxygen atoms from two different ligands, one oxygen atom from the water molecule, and one oxygen atom from the DMA molecule (Figure 1a). Each ligand adopts two coordination modes to link four Zn^2+^ ions, affording a 2D layered framework with an *sql* topology (Figure 1b). The adjacent three layers further form a 2D layered unit by a weak intermolecular interaction (Figure 1c) through O–H···O hydrogen bonds (H···O, 1.834 Å) between the water molecules and the uncoordinated oxygen atom from the ligand to generate a three-dimensional (3D) framework with two channels (Figure 1d). After the removal of the coordinated solvents, interlayer channel I exhibits a cross-sectional size of 11.6 × 7.1 Å^2^, and intralayer channel II exhibits a cross-sectional size of 8.3 × 5.2 Å^2^.

**Figure 2:**
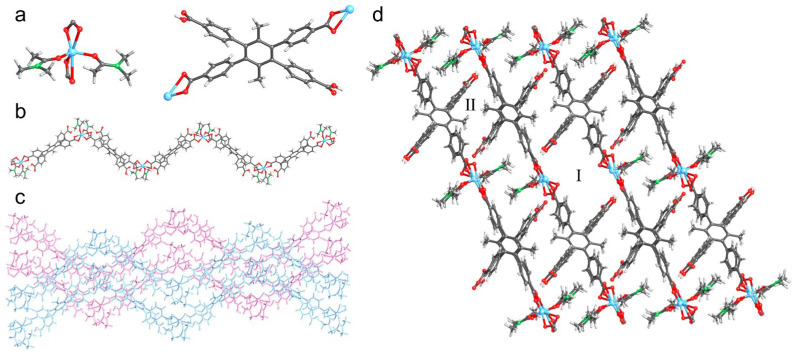
Crystal structure of UPC-97: (**a**) Coordination mode of Zn^2+^ and ligand; (**b**) Infinite S-shaped chain; (**c**) Parallel layered unit of chains in opposite directions; (**d**) 3D framework with two channels. Zn, sky-blue; C, gray; N, green; O, red; and H, pale.

Colorless lamellar crystals of UPC-97 were obtained under similar solvothermal conditions except for the acidic pH value. SCXRD measurements reveal that UPC-97 crystallizes in the triclinic *P*-1 space group. The asymmetric unit contains two Zn^2+^ ions, two partially deprotonated ligands, and four DMA molecules. Each Zn^2+^ ion adopts a distorted octahedron coordination geometry with four oxygen atoms from two different ligands and two oxygen atoms from two different DMA molecules (Figure 2a). Due to the acidic condition, the ligand is partially deprotonated and links two Zn^2+^ ions to form an infinite S-shaped chain (Figure 2b). Adjacent chains are arranged in parallel in opposite directions to afford a layered unit (Figure 2c), which is further connected by O–H···O hydrogen bonds (H···O, 1.813–1.853 Å) between the deprotonated and protonated carboxyl groups from different chains to form a 3D framework with two channels (Figure 2d). After the removal of the coordinated DMA molecules, interlayer channels I and II are observed with cross-sectional sizes of 11.5 × 5.7 and 7.4 × 3.9 Å^2^, respectively.

### 2.2. Structure Characterization

**Figure 3:**
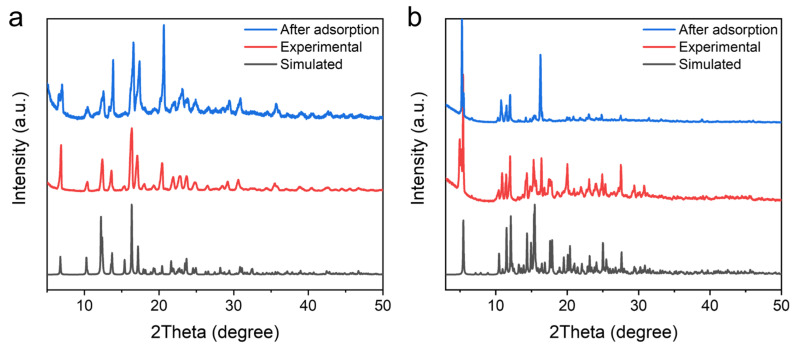
PXRD patterns of (**a**) UPC-96 and (**b**) UPC-97.

The powder X-ray diffraction (PXRD) patterns of UPC-96 and UPC-97 were collected at room temperature to confirm the phase purity (Figure 3). The PXRD patterns of the as-synthesized samples match well with the simulated patterns solved with SCXRD, indicating the correct structures are obtained with good crystallinity and phase purity values. In addition, an elemental analysis further confirms their chemical purity. Anal. calcd for UPC-96 (C_22_H_22_NO_6_Zn): C, 57.22; H, 4.80; and N, 3.03%; found: C, 57.44; H, 4.73; and N, 3.16%. Anal. calcd for UPC-97 (C_88_H_84_N_4_O_20_Zn_2_): C, 64.12; H, 5.13; and N, 3.39%; found: C, 63.93; H, 5.07; and N, 3.46%.

**Figure 4:**
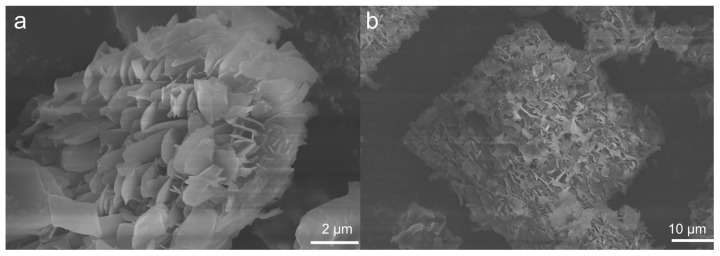
SEM images of (**a**) UPC-96 and (**b**) UPC-97.

Considering that the morphology may significantly impact the adsorption performance, scanning electron microscopy (SEM) images were taken as shown in Figure 4. UPC-96 and UPC-97 both exhibited a uniform flaky morphology, providing surface texture and different levels of microporosity.

**Figure 5:**
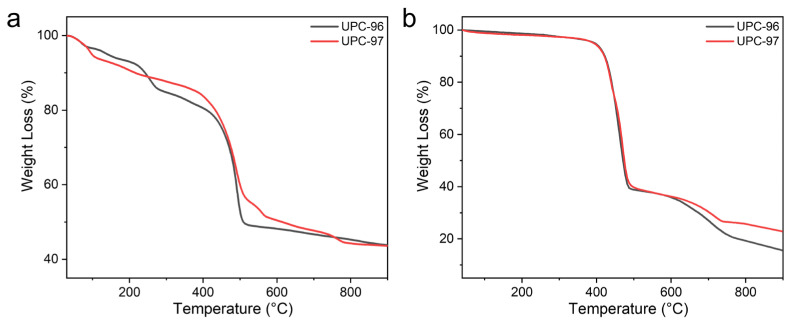
TGA curves of UPC-96 and UPC-97 (**a**) before and (**b**) after activation.

To analyze the thermal stability of UPC-96 and UPC-97, a thermogravimetric analysis (TGA) was performed under a N_2_ atmosphere with a heating rate of 10 °C/min from 40 °C to 900 °C (Figure 5). According to the results of the TGA curves before and after activation, the weight loss of UPC-96 before 240 °C corresponds to the release of coordinated solvent molecules, and UPC-96 remains stable till 400 °C, at which point the framework collapses. In contrast, UPC-97 releases the coordinated solvent molecules before 100 °C and maintains stability until 400 °C, at which point the framework collapses. These investigations confirm their good thermostability.

**Figure 6:**
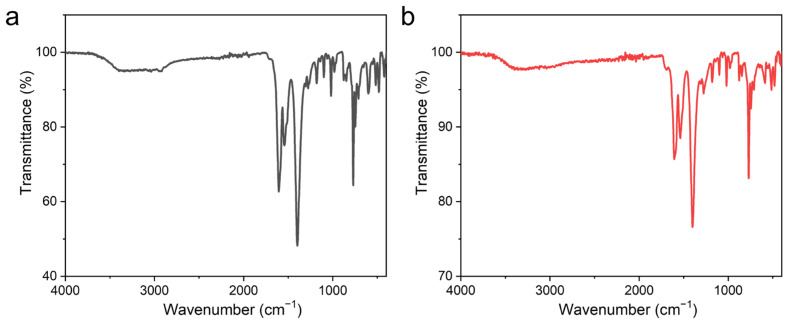
FT-IR spectra of (**a**) UPC-96 and (**b**) UPC-97.

Fourier transform infrared (FT-IR) spectra of UPC-96 and UPC-97 were recorded with KBr pellets (Figure 6). The broad absorption band of UPC-96 at 3400 cm^−1^ is assigned to the hydroxyl stretching vibration of water molecules, which is not observed for UPC-97 without coordinated water molecules. The strong absorption bands of UPC-96 at 1605 and 1390 cm^−1^ and those of UPC-97 at 1605 and 1400 cm^−1^ correspond to the asymmetric and symmetric carboxyl stretching vibrations, respectively.

### 2.3. Gas Adsorption and Separation

**Figure 7:**
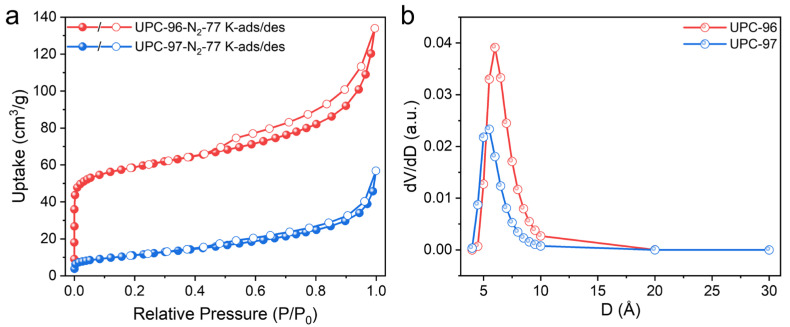
(**a**) N_2_ adsorption/desorption isotherms and (**b**) pore size distribution of UPC-96 and UPC-97.

As mentioned previously, both UPC-96 and UPC-97 possess two kinds of channels with potential for gas adsorption and separation. To confirm the porosity of UPC-96 and UPC-97, N_2_ adsorption/desorption isotherms were collected at 77 K (Figure 7a). UPC-96 exhibited a reversible type I isotherms with a maximum uptake of 133.9 cm^3^/g, which is much higher than that of UPC-97 (56.8 cm^3^/g). The calculated BET surface area of UPC-96 reached 220.2 m^2^/g with a pore volume of 0.19 cm^3^/g, whereas a negligible BET surface area of 37.6 m^2^/g and pore volume of 0.07 cm^3^/g were observed for UPC-97, indicating the significant pore structure difference by solvent regulation. The corresponding pore size distribution was determined by the nonlocal density functional theory (DFT) method (Figure 7b), with the pore size mainly concentrated at 6.0 and 5.5 Å for UPC-96 and UPC-97, respectively.

**Figure 8:**
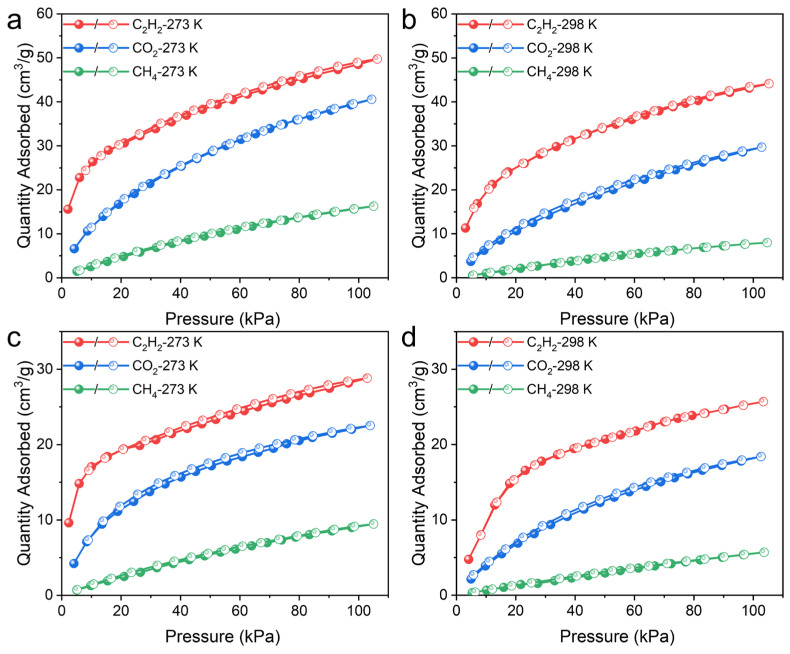
Single-component adsorption/desorption isotherms of C_2_H_2_, CO_2_, and CH_4_: UPC-96 at (**a**) 273 and (**b**) 298 K; UPC-97 at (**c**) 273 and (**d**) 298 K.

Considering the ideal porosity and suitable pore size, the potential of UPC-96 and UPC-97 in C_2_H_2_/CO_2_ and CO_2_/CH_4_ separation was investigated. Single-component adsorption/desorption isotherms of C_2_H_2_, CO_2_, and CH_4_ were measured at 273 and 298 K (Figure 8). As expected, the adsorption capacity follows the order of C_2_H_2_ > CO_2_ > CH_4_, and UPC-96 presents a much higher uptake than UPC-97. The maximum quantities of C_2_H_2_ adsorbed reached 49.7 and 44.1 cm^3^/g for UPC-96 at 273 and 298 K, whereas those for UPC-97 were only 28.8 and 25.7 cm^3^/g at 273 and 298 K, respectively. For CO_2_, the uptake capacities were 40.5 and 29.6 cm^3^/g for UPC-96 at 273 and 298 K, respectively, which were higher than those obtained for UPC-97 (22.5 and 18.4 cm^3^/g). Notably, the CO_2_ uptake capacity of UPC-96 exceeds many reported MOF adsorbents, such as ZNU-11 (22.5 cm^3^/g) [40], BSF-10 (25.8 cm^3^/g) [41], and SNNU-27-Cd (29.5 cm^3^/g) [42]. In contrast, both MOFs exhibited negligible CH_4_ uptake, with the maximum amounts of 16.2 and 7.9 cm^3^/g for UPC-96 and 9.4 and 5.7 cm^3^/g for UPC-97 at 273 and 298 K, respectively. In spite of its low specific surface and porosity values, UPC-97 exhibited a considerable adsorption capacity for C_2_H_2_ and CO_2_ and a negligible adsorption capacity for CH_4_, which may be due to the fact that the kinetic diameter of C_2_H_2_ and CO_2_ is smaller than that of N_2_ (3.64 Å) and CH_4_ (3.8 Å). In addition to the molecular sieving effect, the differential interaction between different gases and MOFs can also explain the different adsorption capacities. The highest C_2_H_2_ capacity could be attributed to the framework and C_2_H_2_ molecules having stronger host–guest interactions than CO_2_ and CH_4_.

**Figure 9:**
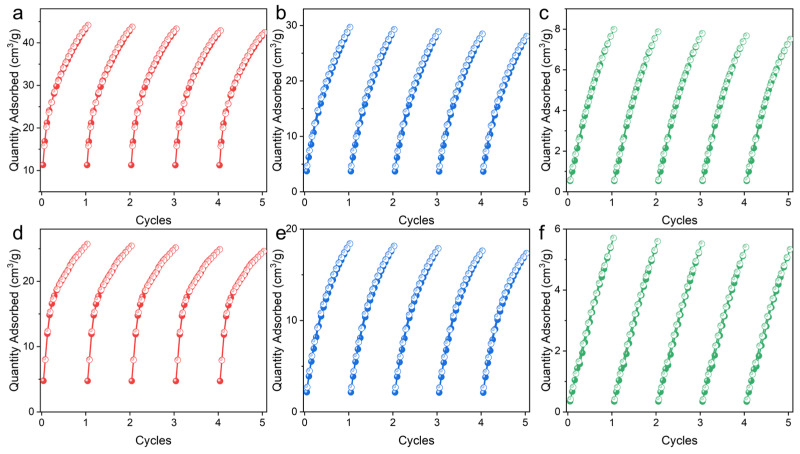
Cycling adsorption/desorption isotherms at 298 K: UPC-96 for (**a**) C_2_H_2_, (**b**) CO_2_, and (**c**) CH_4_; UPC-97 for (**d**) C_2_H_2_, (**e**) CO_2_, and (**f**) CH_4_.

Cycling stability is an important evaluation indicator for adsorbents in practical applications. The repeatability of UPC-96 and UPC-97 was tested for five cycles without a reactivation process at 298 K (Figure 9). For UPC-96, there are only 3.8%, 5.3%, and 6.0% decreases in the adsorption capacity of C_2_H_2_, CO_2_, and CH_4_, respectively; for UPC-97, there are only 4.2%, 5.7%, and 6.5% decreases in the adsorption capacity of C_2_H_2_, CO_2_, and CH_4_, respectively, indicating their good reproducibility. Meanwhile, the PXRD patterns of UPC-96 and UPC-97 after adsorption match well with the original patterns (Figure 3), suggesting their good stability.

**Figure 10:**
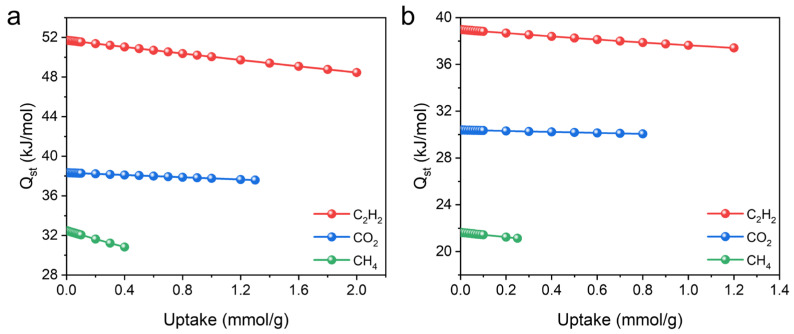
Adsorption enthalpy of (**a**) UPC-96 and (**b**) UPC-97 for C_2_H_2_, CO_2_, and CH_4_.

To evaluate the affinity between the framework and gas molecules, the Virial equation was employed to calculate the adsorption enthalpy (*Q_st_*). In both MOFs, the *Q_st_* value follows the same order as the adsorption capacity, indicating stronger interactions with C_2_H_2_ molecules. At near-zero coverage, the *Q_st_* values of UPC-96 reached 51.7, 38.3, and 32.4 kJ/mol for C_2_H_2_, CO_2_, and CH_4_, respectively, which are higher than those for UPC-97 (38.9, 30.3, and 21.6 kJ/mol) (Figure 10). It is worth noting that the *Q_st_* values of CO_2_ are comparable to many MOFs, such as ZNU-11 (33.9 kJ/mol) [40], BSF-10 (27.4 kJ/mol) [41], and SNNU-27-Cd (24.6 kJ/mol) [42], suggesting a much stronger host–guest interaction between the framework and CO_2_. The high *Q_st_* values of UPC-96 and UPC-97 could be attributed to the abundant open metal sites after the removal of the coordinated solvent molecules [43].

**Figure 11:**
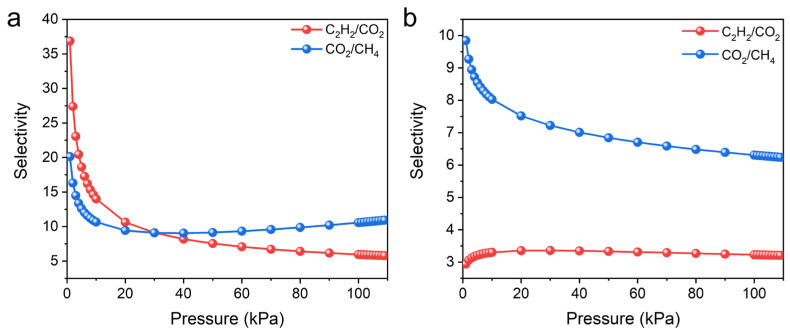
IAST selectivity of (**a**) UPC-96 and (**b**) UPC-97 for equimolar C_2_H_2_/CO_2_ and CO_2_/CH_4_ mixtures at 298 K.

To predict the separation performance, the ideal adsorbed solution theory (IAST) was used to calculate the equimolar C_2_H_2_/CO_2_ and CO_2_/CH_4_ selectivity. In agreement with previous experimental results, UPC-96 exhibited a better separation performance than UPC-97. At 298 K, UPC-96 showed good C_2_H_2_/CO_2_ and CO_2_/CH_4_ selectivity values of 5.7 and 10.9, respectively, whereas C_2_H_2_/CO_2_ and CO_2_/CH_4_ selectivity values only reached 3.2 and 6.2 for UPC-97 (Figure 11). The C_2_H_2_/CO_2_ selectivity is on par with that of some reported adsorbents, such as SNNU-27-Fe (2.0) [42], SIFSIX-Cu-TPA (5.3) [44], and BSF-10 (5.86) [41], while the CO_2_/CH_4_ selectivity surpasses that of MOF-303(Al) (4) [45], PCN-222 (4.3) [46], and Fe-dbai (7.5) [47]. The significant difference between UPC-96 and UPC-97 in adsorption capacity, *Q_st_* value, and IAST selectivity suggests the important role of solvent regulation in the optimization of gas adsorption and separation performance.

## 3. Materials and Methods

### 3.1. Materials and Instruments

All reagents were commercially available and used without further purification.

^1^H nuclear magnetic resonance (NMR) spectrum was obtained using an Inova 500 MHz spectrometer (Inova, Falls Church, VA, USA). Powder X-ray diffraction was carried out on a Bruker D8-Focus Bragg-Brentano X-ray powder diffractometer (Bruker, Billerica, MA, USA) equipped with a Cu sealed tube at 40 kV and 15 mA. Elemental analysis was conducted on a Vario EL III elemental analyzer (Vario, Bohmte, Germany). Scanning electron microscopy images were taken with Hitachi JSM-7500F (Hitachi, Tokyo, Japan) scanning electron microscope. Thermogravimetric analysis was performed using a Mettler Toledo TGA/DSC1 instrument (Mettler Toledo, Columbus, OH, USA) under a static N_2_ atmosphere with a heating rate of 10 °C/min in the range of 40–900 °C. Infrared spectroscopy spectrum was determined using a Nicolet 330 FTIR spectrometer (Nicolet, Mountain, WI, USA) within 4000–400 cm^−1^ region. Gas adsorption measurements were conducted using a Micrometritics ASAP 2020 surface area analyzer (Micrometritics, Norcross, GA, USA).

### 3.2. Synthesis of Ligand

**Figure 12:**
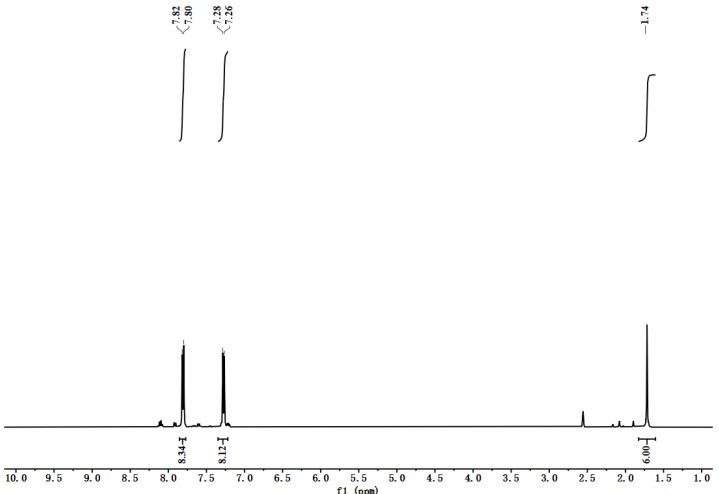
^1^H NMR spectrum of TCPB-Me.

TCPB-Me was synthesized according to the previous literature [48]. ^1^H NMR spectrum proved the good purity of ligand (Figure 12). ^1^H NMR (500 MHz, DMSO-d_6_): δ = 7.82 (d, 8H), 7.28 (d, 8H), 1.74 (s, 6H).

### 3.3. Synthesis of MOFs

UPC-96: A mixture of TCPB-Me (5.0 mg) and Zn(NO_3_)_2_·6H_2_O (10.0 mg) in DMA/H_2_O (5 mL, *v*/*v* = 1/1) was placed into a glass vial (10 mL) and heated at 100 °C for 40 h. The vial was then cooled to room temperature at a rate of 5 °C/h. The obtained colorless rod-shaped crystals were filtered, washed with DMA, and dried in air.

UPC-97 was synthesized with the same method as that for UPC-96 except for the addition of HNO_3_/H_2_O (80 μL, *v*/*v* = 1/10), and colorless lamellar crystals were obtained.

### 3.4. Single-Crystal X-Ray Diffraction

The as-synthesized crystals were taken from the mother liquid without further treatment, transferred to oil, and mounted onto a loop. The data were collected using an Agilent Technologies SuperNova diffractometer equipped with graphite monochromatic Cu Kα radiation (λ = 1.54184 Å). With the help of Olex2 (Version: Olex2-1.5), the structure was solved with the Superflip structure solution program using charge flipping and refined with the ShelXL (Version: ShelXL-2019/3) refinement package using least squares minimization. The structure was treated anisotropically, whereas the hydrogen atoms were placed in calculated ideal positions and refined depending on their respective nonhydrogen atoms. Table 1 shows the crystallographic data and refinement details.

### 3.5. Gas Adsorption Measurements

The activated samples were prepared by immersing the as-synthesized MOFs in chromatography-grade methanol and dichloromethane for solvent exchange followed by activation under vacuum for 8 h at 373 K. Gas adsorption experiments containing C_2_H_2_, CO_2_, and CH_4_ at 273 and 298 K and N_2_ at 77 K were performed by using ASAP-2020 surface area analyzer (Micrometritics, Norcross, GA, USA). Liquid nitrogen bath was used to stabilize the temperature at 77 K, whereas other test temperatures were maintained via a circulating water bath.

### 3.6. Isosteric Heat of Adsorption

A Virial equation comprising the temperature-independent parameters *a_i_* and *b_j_* was employed to calculate the enthalpies of adsorption for C_2_H_2_, CO_2_, and CH_4_ in UPC-96 and UPC-97, which were measured at 273 and 298 K.

$$\ln P=\ln N+\frac{1}{T}\sum_{i}^{m}a_{i}N_{i}+\sum_{j}^{n}b_{j}N_{j}$$

$$Q_{st}=-R\sum_{i=0}^{m}a_{i}N_{i}$$

Here, *P* is the pressure expressed in mmHg, *N* is the amount absorbed in mmol/g, *T* is the temperature in K, *a_i_* and *b_j_* are virial coefficients, and *m, n* represent the number of coefficients required to adequately describe the isotherms (herein, *m* = 5 and *n* = 2). *Q*_st_ is the coverage-dependent isosteric heat of adsorption and *R* is the universal gas constant.

### 3.7. IAST Selectivity

Before estimating the selectivity for binary gas mixture, the single-component gas adsorption isotherms were first fitted to a dual-site Langmuir–Freundlich (DSLF) model:

$$q=q_{A,sat}\frac{b_{A}p^{n_{1}}}{1+b_{A}p^{n_{1}}}+q_{B,sat}\frac{b_{B}p^{n_{2}}}{1+b_{B}p^{n_{2}}}$$

where *q* is the amount of adsorbed gas (mmol/g), *p* is the bulk gas-phase pressure (kPa), *q_sat_* is the saturation amount (mmol/g), *b* is the Langmuir–Freundlich parameter (kPa^−1^), and *n* is the Langmuir–Freundlich exponent (dimensionless) for two adsorption sites A and B, indicating the presence of weak and strong adsorption sites. *b_A_* and *b_B_* are both temperature-dependent.

$$b_{A}=b_{A0}\exp\left(\frac{E_{A}}{RT}\right);\;b_{B}=b_{B0}\exp\left(\frac{E_{B}}{RT}\right)$$

The adsorption selectivity *S_ads_* was calculated by ideal adsorbed solution theory as follows:

$$S_{ads}=\frac{q_{1}/q_{2}}{p_{1}/p_{2}}$$

where *q_1_* and *q_2_* are the molar loadings in the adsorbed phase at equilibrium with the bulk gas phase; *p_1_* and *p_2_* are partial pressure.

## 4. Conclusions

In conclusion, we reported the solvent regulation of pH using the solvothermal method to synthesize two layered Zn-MOFs, UPC-96 and UPC-97. UPC-96 with a completely deprotonated ligand was obtained without the addition of acid, whereas UPC-97 with a partially deprotonated ligand was obtained by adjusting the pH value of solvents to be more acidic. The solvent regulation brought about quite different structures, in which UPC-96 consisted of 2D layers of *sql* topology, and UPC-97 was composed of infinite S-shaped chains. The significant differences in structure thus resulted in differences in their gas adsorption and separation performance. UPC-96 performed better than UPC-97 in BET surface area, pore volume, adsorption capacity, enthalpy, and selectivity. This work provides guidance for the regulation of MOFs’ structure to effectively optimize their specific gas separation performance.

## Figures and Tables

**Table 1:** Crystal data of UPC-96 and UPC-97.

Compound	UPC-96	UPC-97
CCDC	2413283	2413284
Formula	C_22_H_22_NO_6_Zn	C_88_H_84_N_4_O_20_Zn_2_
Formula weight	461.77	1648.33
Temperature/K	294.5 (7)	295.0 (5)
Crystal system	monoclinic	triclinic
Space group	*P*2_1_/c	*P*−1
a/Å	13.0452 (5)	14.6040 (4)
b/Å	9.7574 (4)	16.7263 (5)
c/Å	17.2190 (7)	16.9432 (5)
α/°	90	92.660 (3)
β/°	90.930 (4)	92.832 (2)
γ/°	90	104.788 (3)
Volume/Å^3^	2191.46 (16)	3989.3 (2)
Z	4	2
ρ g/cm^3^	1.400	1.372
μ/mm^−1^	1.876	1.370
F(000)	956.0	1720.0
2θ range for data collection	6.776 to 141.326	7.184 to 133.196
Index ranges	−14 ≤ h ≤ 15	−9 ≤ h ≤ 17
	−11 ≤ k ≤ 9	−19 ≤ k ≤ 19
	−21 ≤ l ≤ 18	−20 ≤ l ≤ 19
Reflections collected	8076	25199
R_int_	0.0649	0.1916
Data/restraints/parameters	4080/511/278	13641/0/1047
Goodness-of-fit on F^2^	1.609	1.310
Final R indexes [I ≥2σ (I)]	R_1_ = 0.1342	R_1_ = 0.1340
	wR_2_ = 0.3930	wR_2_ = 0.3055
Final R indexes [all data]	R_1_ = 0.1507	R_1_ = 0.1779
	wR_2_ = 0.4105	wR_2_ = 0.3339
Largest diff. peak/hole/eÅ^−3^	1.96/−1.33	1.43/−1.36

## Data Availability

The data presented in this study are available on request from the corresponding author. The original contributions presented in this study are included in the article/Supplementary Materials.

## Supplementary Materials

CCDC 2413283-2413284 contains the supplementary crystallographic data for this paper. These data can be obtained free of charge via www.ccdc.cam.ac.uk/data_request/cif, accessed on 4 March 2025, by emailing data_request@ccdc.cam.ac.uk, or by contacting The Cambridge Crystallographic Data Centre, 12 Union Road, Cambridge CB2 1EZ, UK; fax: +44 1223 336033.
